# Postgraduate pariah

**DOI:** 10.1016/S1808-8694(15)30048-3

**Published:** 2015-10-19

**Authors:** Silvio Caldas Neto

Medical residency (MR) is known to be the best means to train a specialist. It is rare to have a physician working as a non-specialist; and even at times like today when we try to value a physician’s training as a general practitioner and numerous Family Practice Residency programs are cropping up around the country, it is clear that MR becomes a postgraduation program which is almost mandatory to physicians.

Notwithstanding, despite its huge importance in our professional training, it has faced problems everywhere it exists, specially in University settings, accruing from the sui generis position it has within medical school flowcharts. Stating that MR is a program to train professionals, Universities are unable to place it as an academic postgraduation program, and although it is under the umbrella of the Postgraduation and Research Departments, MR ends up being left behind, as far as attention is concerned, when compared to master’s, doctorates and even specialization programs, certainly because it is a program that exists exclusively in the medical sphere. It may also be that it implies costs, while the other modes do not. It is then left at the mercy of university hospitals that depend on the Government’s Healthcare Program, facing increasing and historical financial hurdles which already seem unsurmountable. We have this clear idea that MR represents a misunderstood intruder for the University (specially for non-medical managers, who conveniently ignore it).

It is difficult to include MR activities in the official work schedule of our faculty, and not counting this work load reflects on the decisions related to work spot distribution in faculty openings and budget distribution. The non-medical representatives (who do not understand the meaning of MR) at the Universities’ collegiate bodies outnumber the physicians, what makes it even harder to work in trying to bring appreciation to MR programs.

It is true that MRs are professional training programs, however it is far from being just that. Resident physicians bear a huge load of learning and scientific activities. It bears the largest research activities work force in Medicine, since there are not many institutions that offer broad stricto sensu postgraduation programs. Moreover, through seminars and contact with postgraduation students, they have in MR at least a partial academic initiation. A Master’s Degree Program is but a professional scientific and academic initiation. If one only looks at the papers published in most medical journals or presented in conventions, one can see that residents are responsible for a great share of medical scientific production. Notwithstanding, the role of the resident physician in providing medical care to the community is indisputable.

Therefore, we as physicians should put up an effort within the academic and scientific communities in an attempt to value MR as a postgraduation program similar to that of a Master’s Degree. It is high time we convinced federal teaching agencies of the need and urgency of a policy to create new MR programs all over the country and of a continuous modernization of teaching hospitals, so that we may offer society more qualified personnel and state of the art clinical research.

